# Novel regulation of the eEF2K/eEF2 pathway: *prospects of ‘PQBP1 promotes translational elongation and regulates hippocampal mGluR-LTD by suppressing eEF2 phosphorylation’*

**DOI:** 10.1093/jmcb/mjab017

**Published:** 2021-03-18

**Authors:** Yuqian Shen, Junhai Han, Zi Chao Zhang

**Affiliations:** 1 School of Life Science and Technology, Key Laboratory of Developmental Genes and Human Disease, Southeast University, Nanjing 210096, China; 2 Co-innovation Center of Neuroregeneration, Nantong University, Nantong 226001, China

**Figure 1:**
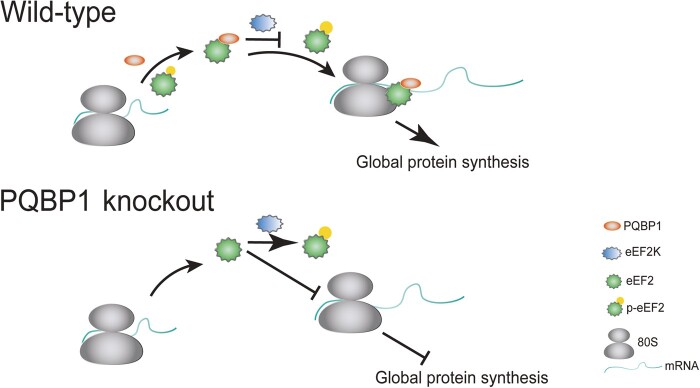
Illustration of the proposed working model. PQBP1 (orange) directly interacts with eEF2 (green) and protects it from being phosphorylated by eEF2K. This interaction facilitates eEF2 binding to 80S ribosome (gray) and promoting elongation along the mRNA (line).

Protein synthesis involves initiation, elongation, and termination. A number of studies show that translation elongation is highly regulated and is the most energy-intensive process, which controls the efficiency and accuracy of protein synthesis. In the elongation process, eukaryotic elongation factor 2 (eEF2) binds to the ribosome to induce its conformation change and promotes the translocation of the tRNAs by hydrolyzing guanosine triphosphate, which then allows the next AA-tRNA to enter the A site for translation elongation. The activity of eEF2 is negatively regulated by its kinase eEF2K that phosphorylates eEF2 at threonine-56 (Thr56). Dysregulation of elongation is the core of the pathogenesis of tumorigenesis and neurodegeneration. Thus, eEF2/eEF2K as a potential therapeutic target has attracted more and more attention (Knight et al., 2020). In a recently published work, we identified that the intellectual disability related protein, polyglutamine-binding protein 1 (PQBP1), is a novel regulator that binds directly with eEF2 and affects its phosphorylation (Figure 1; Shen et al., 2021).

Renpenning syndrome is a group of X-linked intellectual disability disease (XLID) caused by mutations in the human *PQBP1* gene (Kalscheuer et al., 2003; Stevenson et al., 2005). PQBP protein is mainly located in the nucleus and is generally believed to play an important role in transcription and mRNA splicing (Okazawa, 2018). However, previous studies also show that the cytoplasmic PQBP1 associates with and regulates the translation of specific mRNAs (Kunde et al., 2011; Wan et al., 2015). Our study revealed that the cytoplasmic PQBP1 directly binds to nonphosphorylated eEF2 at a linear peptide around Thr56 through its WW domain, protecting eEF2 from being phosphorylated by eEF2K, thus promoting global protein synthesis (Figure 1; Shen et al., 2021). We elucidate the detailed mechanism of how PQBP1 is involved in protein synthesis and demonstrate that individual domains of PQBP1 have distinct molecular functions in different cellular compartments. This function of PQBP1 in translation may be essential for cells as it is highly conserved. *Drosophila* homolog *dPQBP1* is mainly in the cytoplasm and also associates with the ribosomes through the conserved WW domain (Wan et al., 2015). Although common clinical manifestations are present in all patients with PQBP1 mutations, various PQBP1 mutations cause notable variations in the symptoms exhibited by these patients (Stevenson et al., 2005). PQBP1-Y65C mutation from Golabi-Ito-Hall (GIH) family is the first missense mutation found in patients and the only one mutation in the WW domain so far (Lubs et al., 2006). It is suggested that Cys65 residue may be easy to form an intramolecular disulfide bond with Cys60 residue, which reduces the binding of PQBP1 to WBP11/SIPP1 and affects the cleavage of pre-mRNA (Tapia et al., 2010; Sudol et al., 2012). Our data show that the Y65C mutation enhanced its binding ability to eEF2, suggesting that PQBP1-Y65C missense mutation may have some acquired functions. We have generated a *Pqbp1^Y65C^* mouse model for further investigation. Our findings and future work will provide insights into the heterogeneity of Renpenning syndrome.

The WW domain, similar to the SH3 domain, specifically recognizes proline-rich ligands and has been found in many signaling proteins. Espanel and Sudol (1999) classified WW domains based on their proline-based ligand specificity. In that scheme, PQBP1 belongs to the Group II WW domain that preferentially binds to PPLP motifs. One of the interesting findings in our study is that the linear peptide around eEF2 Thr56 interacting with the PQBP1 WW domain is proline-free. In addition, its interaction with the WW domain is affected by the phosphorylation status at Thr56. It is worth characterizing the exact binding motif in eEF2, which will expand our understanding of the diversity of ligands for WW domains and open up a new direction for the intermolecular interaction and signal pathway.

Unlike eEF2 that generally exists in both prokaryotes and eukaryotes, PQBP1 only exists in higher eukaryotes. We suspect that the regulatory mechanism of PQBP1/eEF2/eEF2K is specifically evolved for higher eukaryotes and provides a finer regulatory network for translation to meet various functional requirements of higher eukaryotes. How the PQBP1‒eEF2 interaction responds to different signals and coordinates the growth and development needs further study.

In neuroscience, mounting evidence has shown that dendritically localized eEF2K activity is regulated by neuronal activity and glutamate signaling (Taha et al., 2013; Heise et al., 2014). The eEF2/eEF2K pathway is regulated by different signaling pathways and protein molecules in different physiological environments such as NMDA, mGLu1, mTOR, ERK, growth factors, and Ca^2+^ (Taha et al., 2013). It is associated with different forms of synaptic plasticity (Sutton et al., 2007; Park et al., 2008). Abnormal high levels of phosphorylated eEF2 were detected in neurodegenerative diseases such as Alzheimer's disease, Parkinson's disease, and amyotrophic lateral sclerosis (Delaidelli et al., 2019). We found that the cytoplasmic PQBP1 is crucial for eEF2-dependent mGluR-LTD. PQBP1 suppresses eEF2K-mediated eEF2 phosphorylation that facilitates *de novo* translation of Arc/Arg3.1 during hippocampal mGluR-LTD and regulates mGluR-LTD-associated behaviors (Shen et al., 2021). The balance between PQBP1 and eEF2K in eEF2 phosphorylation ensures the sensitivity of neurons to stimuli and controls protein synthesis in a spatially and temporally limited manner. Thus, PQBP1 may be used for the treatment of neurodegeneration diseases.

Importantly, PQBP1 and eEF2 are ubiquitously expressed, and dysregulation of eEF2 activity is found in different diseases (Liu and Proud, 2016; Beretta et al., 2020; Knight et al., 2020). This newly revealed regulator in eEF2/eEF2K pathway also provides an excellent therapeutic target for various disease conditions, such as neural diseases, virus infection, and cancer.

*[The described work was supported by the National Natural Science Foundation of China (81730034 to J.H.*, *31671045 to Z.C.Z., and 81901287 to Shuting Xia)*, *the Natural Science Foundation of Jiangsu Province (BK20170080 to Z.C.Z.)*, *and Guangdong Key Project (2018B030335001 to J.H.).]*
